# Stanniocalcin 2 drives malignant transformation of human glioblastoma cells by targeting SNAI2 and Matrix Metalloproteinases

**DOI:** 10.1038/s41420-022-01090-6

**Published:** 2022-07-05

**Authors:** Eun-Jin Yun, Donghwi Kim, Jer-Tsong Hsieh, Seung Tae Baek

**Affiliations:** 1 POSTECH Biotech Center, POSTECH, Pohang, Republic of Korea; 2 Department of Life Sciences, POSTECH, Pohang, Republic of Korea; 3 Department of Urology, University of Texas Southwestern Medical Center, Dallas, TX USA; 4 Department of Biotechnology, Kaohsiung Medical University, Kaohsiung, Taiwan Republic of China

**Keywords:** Oncogenes, CNS cancer

## Abstract

Glioblastoma multiforme (GBM) is the most malignant brain tumor and is refractory to conventional therapies. Although previous studies have proposed that the interaction between gene mutations and the external environment leads to the occurrence of GBM, the pathogenesis of GBM is still unclear and much remains to be studied. Herein, we show an association between human glycoprotein stanniocalcin-2 (STC2) and aggressive GBM progression, and demonstrate the underlying mechanism. Elevated STC2 expression and secretion greatly increase GBM cell growth and invasive phenotypes. Mechanistically, both, conditioned media (CM) containing STC2 and recombinant STC2, can induce the transformation of GBM cells into more malignant phenotypes by upregulating the expression of the epithelial-mesenchymal transition transcription factor, snail family transcription repressor 2 (SNAI2) as well as matrix metalloproteinases (MMPs). Moreover, we further demonstrate that the oncogenic function of STC2 in GBM is mediated through the MAPK signaling pathway. Collectively, these results identify the mechanism of STC2 targeting SNAI2 and MMPs through the MAPK pathway in GBM, and provide insights into a potential therapeutic strategy for GBM.

## Introduction

Glioblastoma multiforme (GBM) represents a class of high-grade malignant neoplasms and is the most aggressive brain tumor [1]. Although advances have been made in GBM treatment, including radiation therapy and novel chemotherapeutics following surgical resection, the patient survival time after initial diagnosis has not increased significantly [2, 3]. Over the past decades, concomitant therapy using surgical resection followed by radiotherapy and administration of the methylation agent temozolomide (TMZ) has been the standard care for GBM patients. However, no new treatment has been discovered to improve the median survival and the quest for more effective treatments remains a major goal for GBM research [2]. This lack of progress is due to the complexity of GBM, its extreme heterogeneity, and extraordinary plasticity [4]. As with other types of cancer, specific alterations in oncogenes or tumor suppressor-genes are required during GBM tumorigenesis and progression, and there is substantial evidence that many oncogenes are involved in the progression of GBM [5, 6]. However, little is known about the dysregulated pathways and the mechanisms by which GBM transforms into malignant phenotypes. Thus, the identification of new oncogenes and elucidation of the molecular mechanisms underlying malignant transformation of GBM are essential for the development of tailored therapeutic strategies to the needs of individual patients.

Stanniocalcin-2 (STC2), a member of the stanniocalcin family, is a secreted glycoprotein that is expressed in a variety of tissues and has been implicated in physiological functions, such as calcium and phosphate homeostasis, metabolism, reproduction, and development [7–9]. Additionally, a series of recent studies identify STC2 to be involved in cancer development [10–14]. The expression of STC2 is elevated in several types of cancers, including neuroblastoma [10], prostate [11], ovarian [13], and colon cancer [14], suggesting its potential role in development and progression of cancers. However, the clinical significance and the mechanism by which STC2 plays a role in cancer remain unclear and need to be further elucidated.

This study demonstrates that increased expression of STC2 in GBM cell lines results in increased secretion, which promotes growth and malignancy of neighboring cells. Mechanistically, STC2 regulates the expression of signal family transcription repressor 2 (SNAI2) and matrix metalloproteinases (MMPs) through the MAPK signaling pathway during the malignant transformation of GBM. Therefore, this study demonstrated that STC2-MAPK-SNAI2 signaling axis which could provide insight for the development of potential therapeutics for GBM.

## Results

### Elevated expression of STC2 in malignant GBM

**Fig. 1:**
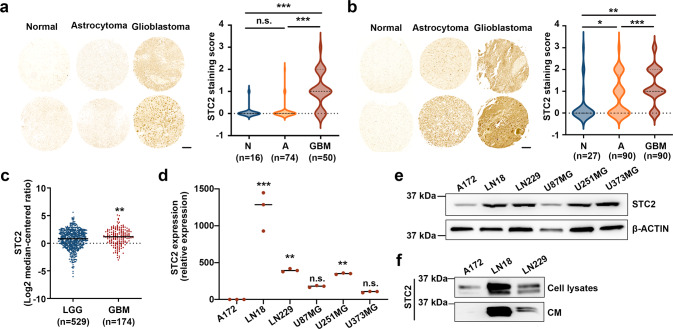
Aggressive GBM expresses higher level of STC2. **a**, **b** Representative tissue microarray data of STC2 in clinical specimens (**a**, GL2082; **b**, GL208, Biomax). The scale bar represents 100 µm. STC2 staining score from 0-4 were analyzed in different tissues. N, normal tissues; A, astrocytoma; GBM, glioblastoma. One-way ANOVA, *p < 0.05, **p < 0.01, ***p < 0.001, n.s., no significance. **c** The levels of *STC2* mRNA expression in low grade glioma (LGG) versus glioblastoma (GBM) tissues obtained from TCGA Research Network were analyzed. Unpaired t-test, **p < 0.01. **d** The mRNA expression of *STC2* in various glioblastoma cell lines were analyzed. After normalizing with 18 S rRNA in each sample, the relative mRNA levels were calculated using A172 = 1. Means ± SD; n = 3 biological replicates. Oneway ANOVA, **p < 0.01, ***p < 0.001, n.s., no significance. **e**, **f** Representative images of Western blotting against STC2 and β-ACTIN in whole lysates of glioblastoma cell lines (**e**) and concentrated culture media (Conditioned media CM) (**f**).

STC2 is widely expressed in human tissues, such as heart, lung, pancreas, and spleen [8, 15]. Several reports suggest elevated expression of STC2 in cancer [10, 14, 16]. To explore the association between the expression of STC2 and the grades of brain cancer, we performed a brain cancer tissue microarray analysis using two different panels. In the first panel, i.e., GL208, the expression of STC2 was found to be highly elevated in GBM tissues compared to normal tissues or astrocytoma tissues (Fig. 1a). In the second panel, i.e., GL2082, the expression of STC2 was found to be elevated in astrocytoma tissues (grade 2-3) compared to normal tissues, and majority of GBM tissues (grade 4) were found to show a stronger STC2 signal, suggesting a positive association between STC2 expression and cancer grades (Fig. 1b). Consistent with these findings, The Cancer Genome Atlas (TCGA) analysis showed that patients with high-grade GBM exhibited significantly higher levels of *STC2* mRNA than patients with low-grade glioma (Fig. 1c). We then characterized *STC2* expression in a series of human glioblastoma cell lines, including A172, LN18, LN229, U87MG, U251MG, and U373MG. Among these cell lines*, STC2* mRNA was highly expressed in LN18, LN229, and U251MG cells whereas *STC2* showed minimal expression in A172 cells at both mRNA and protein level (Fig. 1d, e). Since STC2 is a secreted, hormone-like protein, we additionally analyzed conditioned media (CM) from the cell lines to compare secreted STC2 in the extracellular space. Consistent with the expressions in intracellular compartments, the results showed STC2 expressed at low level in A172 whereas abundant in LN18 cell lines (Fig. 1f). In a previous study, we revealed A172 cells to be chemo-sensitive and less aggressive than LN18 and LN229 [17]. These results together indicate the association between increased expression and secretion of STC2 and higher grade of cancer.

### Malignant transformation of GBM cell lines by STC2

**Fig. 2:**
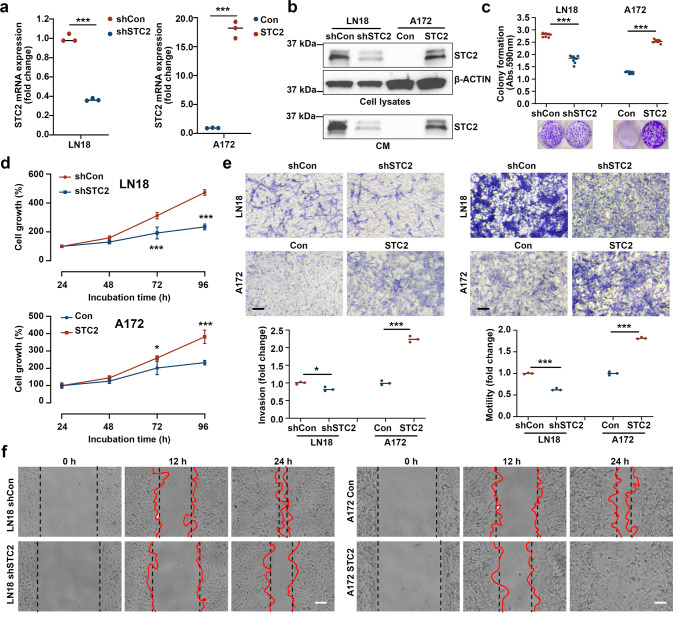
Overexpression of STC2 results in invasive phenotypes of GBM cell lines. **a**, **b** *STC2* mRNA (**a**) and protein (**b**) expression was validated after modulation of STC2. LN18 cell was transfected with pRS-sh*STC2* to knockdown *STC2* whereas A172 cell was transfected with Myc-DDK-tagged-*STC2* to overexpress *STC2*. Means ± SD; *n* = 3 biological replicates; Student’s two tailed t-test, ***p < 0.001. **c** Cells were seeded to 6-well plates at a density of 1000 cells per well and colony formation was determined after 10 days. Absorbance at 590 nm was measured after dissolving with 10% acetic acid. Means ± SD; *n* = 3 biological replicates; Student’s two tailed t-test, ***p < 0.001. **d** Cell growth rates after STC2 modulation were determined by MTT assay. Means ± SD; *n* = 12 biological replicates. Student’s two tailed t-test, *p < 0.05, ***p < 0.001. **e** In vitro invasion and motility were determined onto Matrigel-coated or non-coated Transwell chambers for 48 h. Scale bar = 100 µm. Means ± SD; *n* = 5 biological replicates; Student’s two tailed t-test, *p < 0.05, ***p < 0.001. **f** In vitro migration was determined for 24 h after STC2 modulation. Scale bar = 100 µm.

To investigate the functional role of STC2 in the aggressive phenotypes of GBM cell lines, STC2 expression was modulated; endogenous STC2 was knocked down in the STC2-high LN18 cell line, and exogenous STC2 was introduced into the STC2-low A172 cell line (Fig. 2a, b). While knockdown of intracellular STC2 in LN18 decreased colony formation, overexpression of STC2 in A172 significantly increased colony formation (Fig. 2c). The acceleration of cell growth by STC2 was further confirmed by the cell survival assay (Fig. 2d). Migration and invasion of cancer cells into the surrounding tissues is a major aspect of malignancy [18]. Overexpression of STC2 in the STC2-low A172 cell line (A172 STC2) induced more malignant phenotypes with increased in vitro invasion and motility, in addition to cell migration (Fig. 2e, f). In contrast, knockdown of STC2 in the STC2-high LN18 cell line (LN18 shSTC2) resulted in reduced cell invasion, motility, and migration (Fig. 2e, f), suggesting the involvement of intracellular STC2 in transforming GBM cells toward malignant phenotypes.

### Paracrine function of STC2 in the malignant transformation of GBM cell lines

**Fig. 3:**
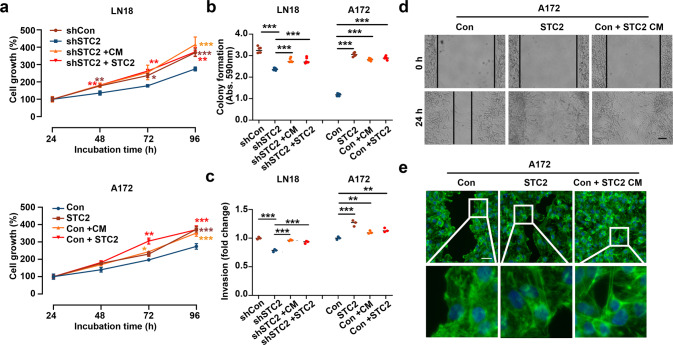
Secreted STC2 induces invasive phenotypes of neighboring GBM cells. **a** Cell growth rates after treatment of recombinant STC2 (100 ng/mL) or STC2-containing CM were determined using MTT assay. Means ± SD; *n* = 12 biological replicates. Student’s two tailed t-test, *p < 0.05, **p < 0.01, ***p < 0.001. Significance was analyzed against shSTC2 in LN18, Con in A172. **b**, **c**) Colony formation (**b**) and in vitro invasion (**c**) were determined after treatment of recombinant STC2 (100 ng/mL) or STC2-containing CM. Absorbance at 590 nm was measured after dissolving with 10% acetic acid. Means ± SD; *n* = 3 biological replicates; One-way ANOVA, **p < 0.01, ***p < 0.001. **d** In vitro migration was determined after treatment of recombinant STC2 (100 ng/mL) or STC2-containing CM. Scale bar = 100 µm. **e** Migrating cells were visualized using fluorescent phalloidin (green) and DAPI (blue) staining. Scale bar = 50 µm.

Modulation of intracellular STC2 expression resulted in corresponding changes in the levels of secreted extracellular STC2 (Fig. 2b). We therefore analyzed the effects of extracellular STC2 on neighboring cells. We collected the STC2-high CM from A172 STC2 and STC2-high LN18 cells. When cultured with 50% CM, both LN18 shSTC2 and A172 Con cells grew two-fold faster compared to their growth on normal media (Fig. 3a). We used recombinant STC2 to analyze the effect of STC2 alone, thus excluding the effect of other factors. Treatment with recombinant STC2 (100 ng/mL) also increased the cell growth rate in a manner similar to that observed in response to STC2 overexpression or STC2-containing CM treatment (Fig. 3a). Furthermore, STC2 CM or recombinant STC2 significantly increased colony formation and in vitro invasion into Matrigel (Fig. 3b, c). Transwell migration assay was performed to visualize the migrating cells with fluorescent phalloidin staining to determine the morphological changes after STC2 stimulation. Although focal adhesion formation and actin cytoskeleton rearrangement did not reveal any significant differences, overexpression of STC2 or STC2-containing CM treatment resulted in a dramatic increase in A172 cell migration (Fig. 3d, e). Altogether, these results suggest that tumor-secreted STC2, as well as intracellular STC2, may induce neighboring cells to become more malignant.

### Aggressive phenotypes induced by increasing expression of SNAI2 and MMPs

**Fig. 4:**
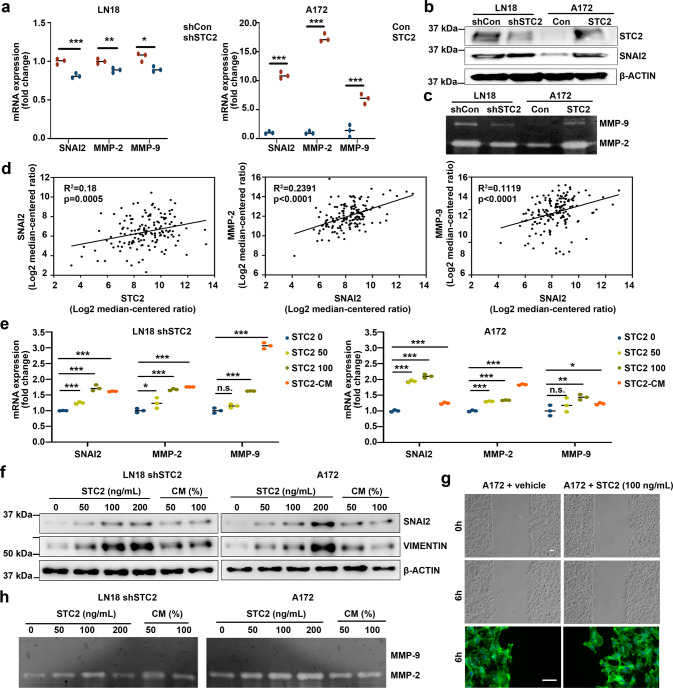
STC2 targets SNAI2 and MMPs. **a** *SNAI2*, *MMP-2*, and *MMP-9* mRNA expressions were validated after modulation of *STC2* in LN18 or A172 cell lines. Means ± SD; *n* = 3 biological replicates; Student’s two tailed t-test, *p < 0.05, **p < 0.01, ***p < 0.001. **b** SNAI2 protein expression was validated after modulation of STC2. **c** Proteolytic activities of MMP-2 and MMP-9 were determined by gelatin zymography after STC2 modulation. **d** The correlation of *SNAI2* expression with *STC2* (left), the correlation of *MMP-2* expression with *SNAI2* (middle), and the correlation of *MMP-9* expression with *SNAI2* (right) were determined in the TCGA data. **e** *SNAI2*, *MMP-2*, and *MMP-9* mRNA expressions were measured after treatment of recombinant STC2 (50, 100 ng/mL) or STC2-containing CM (50%). Means ± SD; *n* = 3; One-way ANOVA, *p < 0.05, **p < 0.01, ***p < 0.001, n.s., no significance. **f** SNAI2 protein expression was validated after treatment of recombinant STC2 (50, 100, 200 ng/mL) or STC2-containing CM (50, 100%). **g** In vitro migration was determined for 6 h after recombinant STC2 (100 ng/mL) treatment. Migrated cells were visualised by staining with fluorescent phalloidin and DAPI. Scale bar = 50 µm. **h** Proteolytic activities of MMP-2 and MMP-9 were determined by gelatin zymography after recombinant STC2 (50, 100, 200 ng/mL) or STC2-containing CM (50, 100%).

STC2 is known to be involved in various physiological processes including regulation of calcium/phosphate transport and homeostasis, by inhibiting the transcription of phosphate transporters [19]. STC2 promotes the proliferation, tumorigenicity, and epithelial-mesenchymal transition (EMT) via PI3K/AKT or AKT-ERK signaling in several types of cancer [13, 20–22]. To investigate the STC2-mediated molecular signaling pathways in the aggressive phenotypes of GBM, we screened the downstream targets of STC2. As STC2 overexpression was associated with increased invasion and migration (Figs. 2, 3), we profiled various EMT factors and identified that the mRNA and protein levels of SNAI2 were consistently elevated in LN18 and A172 STC2 (Fig. 4a, b). In these cell lines, the mRNA levels of extracellular matrix (ECM)-degrading enzymes MMP-2 and MMP-9 were also significantly increased (Fig. 4a). The role of MMPs have been implicated in invasive phenotypes of GBM and degradation of the ECM by proteases, such as MMPs, is an important prerequisite for tumor-cell invasion [23–25]. To further determine whether elevated MMP-2 and MMP-9 expression is linked to changes in enzyme activity, we performed in-gel zymography of STC2-containing CM. As shown in Fig. 4c, knockdown of STC2 in LN18 cells resulted in decreased MMP-2 and MMP-9 activity whereas overexpression of STC2 in A172 resulted in dramatically enhanced activity of both MMP-2 and MMP-9. Supporting these findings, TCGA data analysis revealed positive associations of STC2 with SNAI2, and of SNAI2 with MMPs (Fig. 4d). Together, these results suggest that STC2 enhances invasion and motility by enhancing the expression of SNAI2 and the activity of MMP-2 and MMP-9 in vitro.

We next then confirmed the paracrine effect of STC2 on neighboring cells based on the intracellular regulation axis of STC2. STC2-low cells treated with recombinant STC2 exhibited increased expression of SNAI2, MMP-2, and MMP-9 at mRNA and protein levels in a dose-dependent manner (Fig. 4e, f). Recombinant STC2 thus increased in the migration of A172 cells (Fig. 4g). Similarly, CM containing STC2 also enhanced the expression and activity of SNAI2, MMP-2, and MMP-9, suggesting that secreted STC2 targets the same downstream molecules to induce invasive phenotypes in neighboring GBM cells (Fig. 4e–h).

### Activation of the MAPK pathway by STC2

**Fig. 5:**
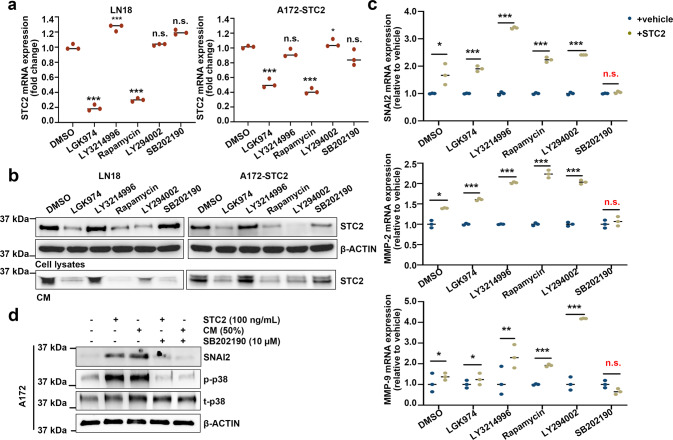
Secreted STC2 regulates SNAI2 and MMPs through p38 MAPK pathway. **a**, **b** LN18 and A172-STC2 cells were treated with small molecule signaling pathway inhibitors (LGK974, 500 nM; LY3214996, 50 nM; Rapamycin, 10 nM; LY294002 1 µM; and SB202190, 10 µM) for 48 h and STC2 expression was detected by real-time PCR (**a**) and Western blot analysis (**b**). Means ± SD; *n* = 3; On-way ANOVA, ***ī< 0.001. **c** Wild-type A172 cells were co-treated with small molecule signaling pathway inhibitors and recombinant STC2 (100 ng/mL) for 48 h and *SNAI2*, *MMP-2*, and *MMP-9* mRNA expressions were determined by real-time PCR. The relative mRNA levels were calculated against vehicle control of each inhibitors. Small molecule inhibitors were added 30 min prior to recombinant STC2 treatment. Means ± SD; *n* = 3; Student’s two tailed t-test, *p < 0.05, **p < 0.01, ***p < 0.001. **d** A172 cells were treated with recombinant STC2 (100 ng/mL) or STC2-containing CM (50%) with or without p38 MAPK inhibitor SB202190 for 48 h. SNAI2 protein expression along with phosphorylated p38 (p-p38) and total p38 (t-p38) proteins were determined by Western blot analysis.

To further investigate the upstream and downstream regulation mechanisms of STC2, we explored several known signaling pathways. LN18 and A172 STC2 cell lines were treated with inhibitors of specific signaling pathways at optimal concentrations to effectively block them without affecting cell survival [17]. After 48 h of treatment, intracellular *STC2* mRNA expression was significantly suppressed by blockage of Wnt/β-catenin pathway (LGK974) or mTOR signaling (Rapamycin) in both cell lines (Fig. 5a). STC2 protein expression was also decreased upon treatment with Wnt/β-catenin or mTOR inhibitors. The PI3K pathway (LY294002) was also observed to play an important role in the protein expression of STC2 (Fig. 5b). Reduced expression of STC2 protein resulting in decreased secretion, and MAPK blockage (SB202190) also suppressed STC2 secretion without affecting STC2 protein expression (Fig. 5b). These results together suggest that a combination of multiple signaling pathways is intricately involved in the regulation of STC2 expression and secretion.

Although receptors for STC2 have not yet been identified [26], we determined the key signaling pathway involved in secreted STC2-induced GBM transformation by using small molecules to block the signaling pathways. Blocking signaling pathways affected downstream targets including *SNAI2*, *MMP-2*, and *MMP-9* mRNA, and the expression of the downstream target genes was significantly increased upon treatment with recombinant STC2 (Fig. 5c). The effect of recombinant STC2 was blocked effectively in cells pretreated with p38 MAPK inhibitor (Fig. 5c). We further confirmed this finding at protein level in A172 cells and found that blockage of p38 prevented SNAI2 expression (Fig. 5d). These results indicate that secreted STC2 regulates the malignant transformation of neighboring GBM cells through the MAPK signaling pathway.

## Discussion

Emerging evidence suggests that the aberrant expression of STC2 in tumor tissues is involved in GBM progression; however, the underlying mechanisms are not fully understood. The relationship between STC2 expression in cancer tissues and the prognostic outcomes is controversial as STC2 functions as an oncogene in some type of cancers including colon, lung, and hepatocellular carcinomas, but a tumor suppressor in other types of cancer, as evidenced by its suppression of breast cancer cell migration and invasion [14, 27–30]. We have focused our studies on elucidating the role and mechanism by which STC2 affects GBM progression, aiming for a comprehensive understanding of the potential role of STC2 as a surrogate marker or therapeutic target for patients with aggressive GBM. In this study, we confirmed the STC2 mRNA level to be significantly elevated in GBM tissues compared to that in normal tissues by analyzing tissue microarray and TCGA data. Overexpression of intracellular STC2 or treatment with recombinant STC2 increased GBM cell proliferation and invasive phenotypes. Our data suggest that STC2 has oncogenic potential that promotes GBM progression, thus making STC2 a potential target for GBM treatment.

Since the identification of human *STC2* cDNA, many structural and molecular studies have demonstrated the function of STC2 indirectly [15, 19, 31, 32]. However, its detailed regulatory mechanisms are not fully understood. In renal cells, STC2 inhibits the promoter activity of type II sodium phosphate transporters, resulting in a reduction in phosphate uptake [19, 33]. STC2 has been implicated in calcium/phosphate regulation based on its ability to enhance inorganic phosphate-induced calcification and limit ectopic calcification [34]. Changes in intracellular Ca^2+^ involved in EMT [35], cancer metastasis [36], and drug-resistance [37]. It has been demonstrated a link between the calcium-related functions of STC2 and cancer [38], however, STC2-overexpressing transgenic mice having normal serum Ca^2+^ and phosphate levels [12], as well as STC2 knockout mice exhibiting no changes in serum Ca^2+^ [39]. In addition, secreted STC is more likely to function locally in an autocrine or paracrine manner, as radiolabelled recombinant STC was reported to be rapidly modified and eliminated [40]. We focused on the autocrine/paracrine functions of STC2 rather than on its involvement in calcium homeostasis, and revealed the EMT transcription factor SNAI2 to be one of the downstream targets of STC2. Treatment with either STC2-containing CM or with recombinant STC2 significantly increased SNAI2 expression to a level comparable to that observed in response to intracellular STC2 overexpression, suggesting the paracrine action of STC2. Identification of receptors for STC2 will facilitate our understanding of the direct regulatory mechanism [26]. We also explored several signaling pathways using small molecule inhibitors. Our results showed that pre-treatment with SB202190, a p38 MAPK inhibitor, blocked the effect of STC2 on SNAI2 expression. This indicates the MAPK signaling is involved in the regulation of SNAI2 expression induced by STC2 in GBM. As the MAPK signaling pathway is modulated by multiple signaling and crosstalk with various signaling pathways [41, 42]. Other signaling pathways known to be involved in the downstream regulation mechanism of STC2 such as PI3K [14], showed no significant effect in our system. A proposed explanation is that this pathway may be involved in the expression or secretion of STC2 upstream rather than downstream. The Wnt/β-catenin, PI3K/Akt, and MAPK pathways affected the expression of the STC2 protein in GBM cells as well as its secretion from the very same cells suggesting a complex regulation involving multiple signaling pathways. Elucidating the precise upstream and downstream regulatory mechanisms of oncogene such as STC2 warrants further investigation as it will open the door to a wider variety of potential targets for treatment.

Lastly, although GBM has been extensively studied, the underlying molecular mechanisms remain elusive and the prognosis of GBM patients is still very poor. Therefore, there is an urgent need for more useful biomarkers to predict the prognosis of GBM. In this aspect, our study indicates that STC2 can serve as a novel biomarker for malignant GBM and provides insights for developing therapeutic strategies against GBM.

## Materials and methods

### Cell lines and reagents

Human glioblastoma cell lines LN18, and A172 were grown in Dulbecco’s Modified Eagle’s Medium (DMEM) (Thermo Fisher Scientific) containing 10% heat-inactivated FBS and antibiotics (penicillin/streptomycin 100 IU/mL, Gibco) at 37 °C in a 5% CO_2_ atmosphere. All cell lines were mycoplasma-free and routinely tested by PCR amplification.

### Plasmid construction and reagents

The human *STC2* expression plasmid (Cat#RC200537, OriGene) and shRNA plasmid kit (Locus ID 8614, Cat#TR309053, OriGene) were transfected using Xfect (Takara Bio Company according to the manufacturer’s protocol. Briefly, Xfect polymer was added into each plasmid (5 µg) in Xfect reaction buffer and incubated for 10 min at room temperature to allow nanoparticle complexes to form. Then, the entire complex solution was added dropwise to cells in 6 well plates. After transfection, cells were selected by independently exposing them to G418 or puromycin for 3–4 weeks. The following primary antibodies were used: rabbit polyclonal anti-STC2 (ab262857, Abcam), rabbit monoclonal anti-actin (Cat#4970, Cell Signaling Technology), anti-SNAI2 (Cat#9585, Cell Signaling Technology), anti-Vimentin (Cat#5741, Cell Signaling Technology). Human recombinant STC2 (Cat#9405-SO) was purchased from R&D Systems. LGK974 (S7143), LY3214996 (S8534), Rapamycin (S1039), LY294002 (S1105), and SB202190 (S1077) were purchased from Selleckchem.

### Tissue microarray (TMA)

Brain primary tumor tissue microarray slides (Cat# GL208 and GL2082) were purchased from US Biomax, Inc. (MD, USA). Formalin-fixed, paraffin-embedded sections were deparaffinized, rehydrated and subjected to heat-induced antigen retrieval (10 mM citrate buffer, pH6.0). Sections were blocked with CAS-Block reagent (Thermo Fisher Scientific, MA, USA), and incubated with STC2 antibody. After blocking endogenous peroxidase activity, immunohistochemistry of STC2 was performed using a VECTASTAIN Elite ABC HRP Kit (Vector Labs, CA, USA) according to the manufacturer’s instruction and counterstained with haematoxylin. Each sample stained with STC2 was scored as negative (0), weak (1), moderate (2), or strong (3) based on the staining intensity.

### RNA preparation and real-time PCR

Total RNA was isolated from cultured cells using ReliaPrep RNA Miniprep Systems (Promega) and 1 μg RNA was reverse transcribed into cDNA using LunaScript (Promega). Real-time PCR was performed using PowerUP SYBR Premix (Applied Bioscience). The relative level of target mRNA was normalized using the delta-delta Ct method and the fold change was determined by normalizing with 18 S rRNA. All experiments were performed at least in triplicate.

### Western blotting

Collected cells were washed with PBS and lysed in RIPA buffer supplemented with protease inhibitor and phosphatase inhibitor. For Western blotting, cell lysates were subjected to electrophoresis on a pre-cast SDS-polyacrylamide gel (4-12%, Bio-Rad). Separated proteins were transferred onto nitrocellulose membranes, blocked with 5% skimmed milk (w/v) for 1 h. Membranes were then incubated with primary antibodies at 4 °C overnight, followed by incubating incubation with the appropriate HRP-conjugated secondary antibodies. Signals were visualised using LAS 4000 (GE Healthcare Life Sciences) after exposing the membrane to the Clarity Western ECL Substrate (Bio-Rad).

### Cell proliferation assay

Cells (5 × 10^3^) were seeded in 96-well plate and incubated for 24–96 h, after which they were indicated with Cellrix viability assay kit (MediFab) for 2 h at 37^°^C, and the absorbance was measured at 450 nm using a Multi-Mode Microplate Reader (BioTek).

### Colony formation assay

Cells (1000 cells/well) were seeded onto 6-well plates and cultured for 10 days. Colonies were fixed and stained with 4% paraformaldehyde in PBS containing 0.02% crystal violet for 30 min. After staining, colonies were gently washed and counted. Absorbance at 590 nm was measured after dissolving the crystal violet with 10% acetic acid.

### In vitro invasion and motility assay

Transwell (24-well insert; pore size 8 μm, Corning) polycarbonate filters (6.5 μm) were coated with 50 μL Matrigel. Cells were resuspended in serum-free media and seeded in the upper chamber (5 × 10^4^ cells/200 μL/chamber). The lower chambers were filled with 500 μL of serum-containing media. After culturing for 48 h, noninvading cells on the upper surface of the membrane were removed using a cotton swab and cells on the lower surface were stained with 1% crystal violet. Five randomly chosen areas were photographed under a microscope and the absorbance was measured at 590 nm was measured after dissolving the crystal violet with 10% acetic acid.

### Migration assay and Phalloidin staining

Cells were resuspended at a density of 5 × 10^5^ cells/mL, and 70 µL of the cell suspension was seeded in a Culture-Insert 2 Well (Cat#81176, Ibidi). After appropriate cell attachment, the silicone insert was gently removed using sterile tweezers. The used plates filled with cell free media were incubated for 24 h and photographed at 0, 6, 12, 24 h time point. After incubation for 24 h, the cells were washed carefully with PBS and fixed in 4% paraformaldehyde. Then, cells were stained with fluorescent phalloidin (Cat# A12379, Invitrogen) and DAPI (Cat# D1306, Invitrogen) in accordance to the manufacturers’ instructions. Migrated cells were photographed using a fluorescence microscope (ECLIPSE Ts2R FL, Nikon).

### Zymography

Gelatin (1 mg/mL) was prepolymerized on 7.5% polyacrylamide gel. Conditioned media (CM) from the cell culture were collected and subjected to electrophoresis under nonreducing conditions to analyzes gelatin degradation. After protein separation, the gel was washed twice with the washing buffer (50 mM Tris-HCl, pH 7.5; 5 mM CaCl_2_; 1 µM ZnCl_2_; 2.5% Triton X-100), and incubated with the incubation buffer (50 mM Tris-HCl, pH 7.5; 5 mM CaCl_2_; 1 µM ZnCl_2_; 1% Triton X-100) at 37 °C for 24 h. Degraded bands were visualised by staining (0.1% Coomassie Blue in 10% acetic acid, 40% methanol) for 30 min followed by the destaining step.

## Supplementary information

Original Western blot

## Data Availability

The data generated in this study are available upon request from the corresponding author.
