# Supplementary figures and images for: Stanniocalcin 2 drives malignant transformation of human glioblastoma cells by targeting SNAI2 and Matrix Metalloproteinases
Source: Cell Death Discov. 2022 Jul 5;8:308. doi: 10.1038/s41420-022-01090-6 (PMC9256701; doi:10.1038/s41420-022-01090-6)

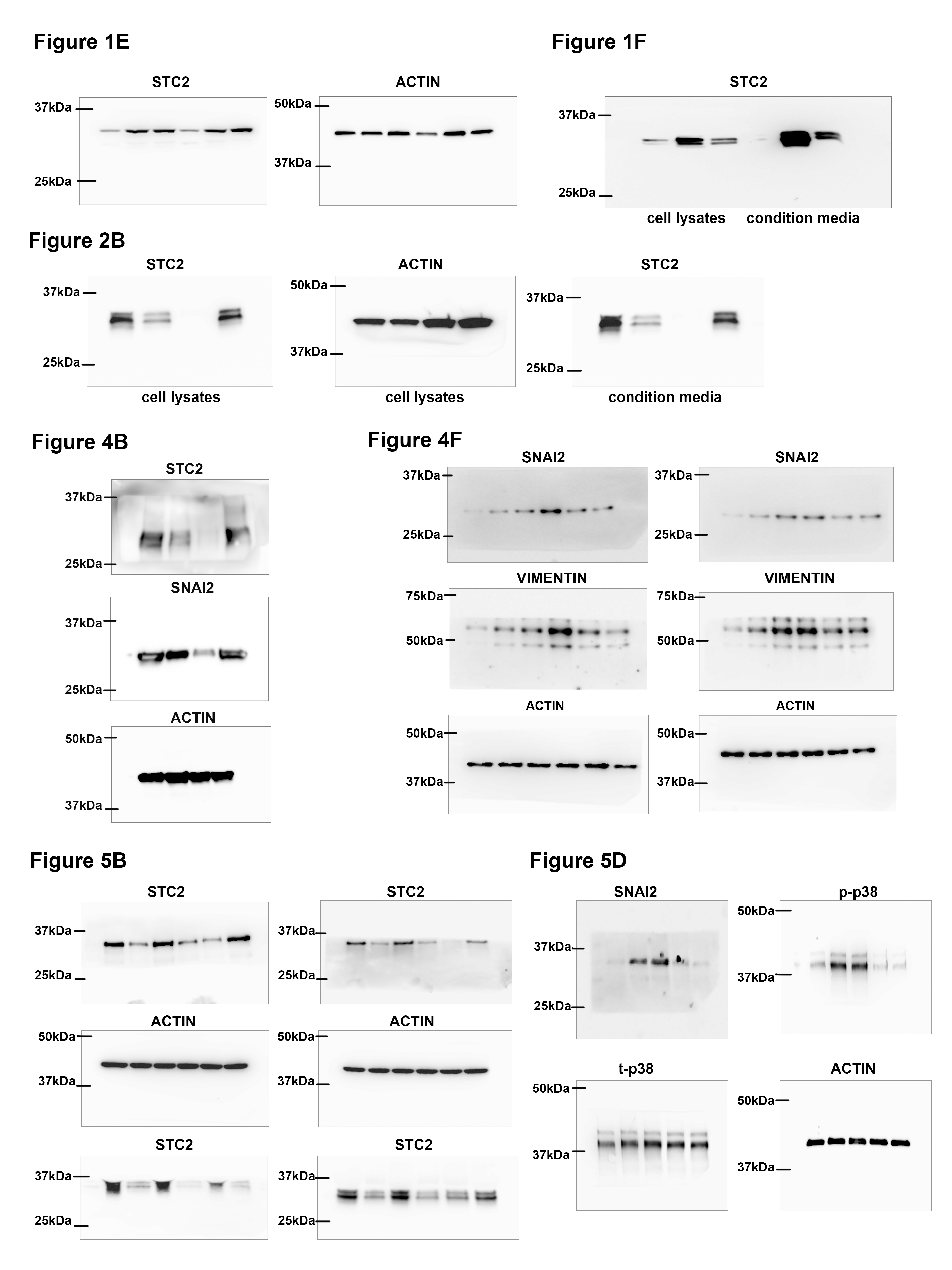

Supplement: Supplementary file 1 — Original Western blot [file 41420_2022_1090_MOESM1_ESM.tif]
